# Genetic variation in salt taste receptors impact salt intake and blood pressure

**DOI:** 10.1038/s41598-022-23827-0

**Published:** 2023-03-10

**Authors:** Noushin Mohammadifard, Faezeh Moazeni, Fatemeh Azizian-Farsani, Mojgan Gharipour, Elham Khosravi, Ladan Sadeghian, Asieh Mansouri, Shahin Shirani, Nizal Sarrafzadegan

**Affiliations:** 1 Isfahan Cardiovascular Research Center, Cardiovascular Research Institute, Isfahan University of Medical Sciences, Isfahan, Iran; 2 Department of Clinical Nutrition, School of Nutrition and Food Science, Food Security Research Center, Isfahan University of Medical Sciences, Isfahan, Iran; 3 Interventional Cardiology Research Center, Cardiovascular Research Institute, Isfahan University of Medical Sciences, Isfahan, Iran; 4 Cardiac Rehabilitation Research Center, Cardiovascular Research Institute, Isfahan University of Medical Sciences, Isfahan, Iran; 5 Heart Failure Research Center, Cardiovascular Research Institute, Isfahan University of Medical Sciences, Isfahan, Iran; 6 Perdiatric Cardiovascular Research Center, Cardiovascular Research Institute, Isfahan University of Medical Sciences, Isfahan, Iran; 7 Hypertension Research Center, Cardiovascular Research Institute, Isfahan University of Medical Sciences, Isfahan, Iran; 8 Department of Cardiology, Tehran University of Medical Science, Dr Ali Shariati Hospital, Tehran, Iran

**Keywords:** Medical research, Molecular medicine

## Abstract

So far, few studies have examined the effect of salt taste receptors genetic variation on dietary intake in the Iranian population. We aimed to evaluate associations between single nucleotide polymorphisms (SNPs) in salt taste receptors’ genes with dietary salt intake and blood pressure. A cross-sectional study was carried out among 116 randomly selected healthy adults aged ≥ 18 in Isfahan, Iran. Participants underwent sodium intake determination by 24-h urine collection, as well as dietary assessment by semi-quantitative food frequency questionnaire and blood pressure measurement. Whole blood was collected to extract DNA and genotype of SNP rs239345 in *SCNN1B* and rs224534, rs4790151 and rs8065080 in *TRPV1* gene. Sodium consumption and diastolic blood pressure were significantly higher in carriers of the A-allele in rs239345 compared to subjects with the TT genotype (4808.4 ± 824.4 mg/day vs. 4043.5 ± 989.3 mg/day; P = 0.004) and 83.6 ± 8.5 mmHg vs. 77.3 ± 7.3 mmHg; *P* = 0.011), respectively. The level of sodium intake was lower in the TT genotype of *TRPV1* (rs224534) than the CC genotype (3767.0 ± 713.7 mg/day vs. 4633.3 ± 793.5 mg/day; *P* = 0.012). We could not find any association between genotypes of all SNPs with systolic blood pressure as well as genotypes of rs224534, rs4790151 and rs8065080 with diastolic blood pressure. Genetic variations can relate with salt intake and consequently may associate with hypertension and finally cardiovascular disease risk in the Iranian population.

## Introduction

Approximately, 1.7 million cardiovascular disease (CVD) deaths in 2010 were attributed to high dietary sodium consumption, accounting for 10% of all CVD deaths. Furthermore, in a recent study by Messerli, it has been shown that sodium intake correlates positively with life expectancy and inversely with all-cause mortality worldwide and in high-income countries claims against dietary sodium intake are a reason of decreasing life span or a risk factor for premature deaths ^1,2^. Both individual- and population-based studies have shown that genetic and environmental factors significantly influence sodium consumption and consequently, the blood pressure (BP) level ^3^. It has been shown that excessive sodium consumption can be attributed to higher preferences for salty foods, which may be linked to genetic factors such as salt taste receptor function ^4^.

The amiloride-sensitive (AS) fibers using the *ENaC* protein moderate sodium taste preference, which typically shows a lower taste concentration threshold. However, the amiloride-insensitive (AI) fibers and hence *TRPV1* can regulate aversive responses to salt concentrations ^5^. Therefore, AS and AI fibers structures facilitate salt taste transduction. It has been shown that SNPs in the *SCNN1B* gene polymorphism of AA/AT, rs239345, coded for ENaC β subunit and *TRPV1* genes including rs239345, rs3785368, rs8065080 are associated with differences in salt taste perception and BP among adults and children ^6,7^. In addition, inter-individual variation sources such as environmental and cultural determinants of dietary intake, may have an important controversial role in salt preference, as well ^8,9^. Consequently, understanding the genetic variation in taste perception may lead to new personalized dietary approaches, which can reduce the risk of CVDs ^10^. There are many controversies on the association of various SNPs in the SLC4A5, SCNN1B and TRPV1 genes like SLC4A5 rs7571842, SLC4A5 rs10177833, SCNN1B rs239345 and TRPV1-rs8065080 with the salt intake, preference and sensitivity along with health markers like SBP and DBP ^6,11–17^. However, studies have mostly been conducted in Caucasians from Europe, US or Canada ^11–14^ and few studies have examined the effect of genetic variations on the sodium intake in diverse populations such as Middle East countries like Iran. Therefore, more research is needed to determine the effect of single nucleotide polymorphisms (SNPs) in different taste receptor genes on taste sensitivity. This study aimed to examine the effect of a set of SNPs on sodium intake, food contribution in sodium intake and BP among Iranian adults.

## Materials and methods

### Design and subjects

The present cross-sectional study consisted of 116 adults aged > 18 living in Isfahan, Iran. Exclusion criteria included diagnosis of diabetes, renal insufficiency, having special dietary regimen, fasting or menstruation (for women) on the day of sampling, using diuretics (because of 24-h urine collection to estimate salt intake) and oral contraceptives, the women who are pregnant and lactating, participants with impaired taste, excessive sweating during an unusually hot day or unusual physical activity and incomplete 24-h urine collection.

Participants were selected using multi-stage random cluster sampling method. One adult person aged > 18 was selected from each household. Considering the minor allele frequency of 26.42 for *SCNN1B* (rs239345), sample size calculated by 106 and after predicting 10% non-response rate, final sample size for this study was 116. They were referred to Isfahan Cardiovascular Research Institute (ICRI) for data collection. Our participation rate was 92%. We obtained written informed consents from all participants. All methods were carried out in accordance with relevant guidelines and regulations (e.g. Helsinki declaration). This study was approved by ethical committee of National Institute for medical research Development (IR.NIMAD.REC.1397.346).

### Data collection

Trained health professionals conducted the detailed interviews to obtain information about participants’ socioeconomic status including education, occupation, demographic characteristics and smoking habit. Physical activities were assessed by means of International Physical Activity Questionnaire (IPAQ) ^18^.

#### Anthropometrics and blood pressure measurements

Trained health professionals measured standing height without shoes and recorded to the nearest 0.5 cm. Body weight was measured for subjects wearing light clothes, without shoes and recorded to the nearest 0.5 kg. Body mass index (BMI) was calculated as weight divided by height squared (kg/m^2^). While subjects were standing, waist circumference (WC) was measured midway between the lower ribs and the iliac crest in a horizontal plane. Hip circumference was also measured at the point yielding the maximum circumference over the buttocks using a non-elastic meter.

BP was measured (twice in each arm) manually with a mercury sphygmomanometer according to standard protocols ^19^, after resting for 5 min in a seated position. The first Korotkoff sound was recorded as the systolic BP (SBP) and the disappearance of the sounds (V phase) was considered as the diastolic BP (DBP). We used the mean BP from the arm with the highest value ^16^.

#### Dietary assessment

Dietary intake was assessed using validated 136-item semi-quantitative food frequency questionnaire (FFQ) ^20^. This FFQ was prepared to determine the amount of sodium intake and food contribution in sodium intake. We calculated the discretionary salt which was added at table and in cooking through questioning about the weight of salt packages, the number of households, and the period of time that each salt package is used ^20^. This FFQ was validated for assessment of sodium intake against two reference methods including twelve 24-h dietary recall which were completed monthly during a year among 113 heathy adults aged ≥ 19 in Isfahan, Iran. They administered two FFQ at the beginning and after 1 year to evaluate the reproducibility. The deattenuated Spearman correlation coefficient between the contribution of food sources of sodium based on the FFQ and 24-h dietary recalls varied from 0.39 for canned food to 0.53 for added salt (P < 0.001). The deattenuated Spearman correlation coefficient between the FFQ and 24-h dietary recall for total sodium intake was 0.65 (P < 0.001). Intraclass correlation coefficient (95% CI) between two FFQ ranged from 0.20 (0.005–0.37) (P = 0.031) for sauces to 0.49 (0.28–0.69) for bread. According to the Bland–Altman plots, we observed an acceptable level of agreement between the two methods for sodium intake ^20^.

All participants were asked to answer how often they had consumed food items over the past year in 9 options including never or rarely (less than 1 per month), 1–3 per month, 1 per week, 2–4 per week, 5–6 per weak, 1 per day, 2–3 per day, 4–5 per day and 6 or more per day. The Iranian Food Consumption Program (IFCP) ^21^ was used to calculate sodium intake and food group servings for all foods reported in the FFQ using the Iranian Food Composition Table ^22^. The FFQ contained four questions about discretionary salt consumption including the salt used at the table; the weight of the salt package used; time taken to consume each salt package and the number of family members ^23^.

#### Urine collection

The morning urine samples were collected on 2 days at 7:00 a.m. (the first sample of the first day was excluded) and was poured into a sterile plastic container labeled with the participants’ ID and a special code. The samples with low volume or lack of proper collection were excluded. The urine samples were collected at home for those that the delivery of their samples was impossible for any reason. Fasting venous blood samples were taken to measure serum biochemical parameters including, fasting blood glucose (FBG), serum albumin level, and lipid profile. Each participant provided two samples for the urinary sodium, potassium and chloride. The mean of 24-h urine sodium excretion was used to estimate daily salt intake. In order to obtain the whole 24-h urinary Na (24hUNa), we multiplied Na concentration by the volume in liters. The urinary sodium, potassium and chloride were measured by emission flame photometry and creatinine (Cr) was measured by the Jaffe method (Technical SMA 12–60) ^24^ in 24-h urine samples. The 24-h urine samples’ completeness was evaluated through the following criteria: Total 24-h urine volume sample ≥ 500 mL, missing no more than 1 void during collection, and collection of ≥ 20-h and 24-h urine creatinine (24hUCr) ≥ 20 mg/dL per kg of body weight in men and ≥ 15 mg/mL per kg of body weight in women aged < 50 and 24hUCr ≥ 10 mg/dL per kg of body weight in men and ≥ 7.5 mg/mL per kg of body weight for men and women aged ≥ 50 ^25^.

### Single nucleotide polymorphisms selection and genotyping

A PubMed SNP search was conducted for the following genes associated to taste detection. The selected SNPs were filtered by global minor allele frequency (MAF), and SNPs with a minor allele frequency below 5% were removed. The resulting SNPs were filtered using HaploView 4.2 software to obtain tag SNPs (tSNPs). Each tSNP was considered independent due to low linkage disequilibrium (r2 < 0.05). *SCNN1B* rs239345 (MAF = 0.2642) *and TRPV1* gene rs4790151 (MAF = 0.2138), rs224534 (MAF = 0.378) and rs8065080 (MAF = 0.3177). Dias et al.’s study showed that TRPV1 receptor might have an important role in salt preference ^6^. Rs8065080, in the TRPV1 gene, affect functional activity of TRPV1 receptors and be involved in different risk factors and pain conditions ^26^. González-Mercado et al.’s study showed that four SNPs including rs224534 and rs8065080 are located in this gene as haplotype ^24^. Previous studies reported the role of TRPV1-rs8065080 polymorphism in ion channel function, rs224534 and two other variants in higher sodium intake ^27,28^. There is little evidence about rs3785368 variant to suggest its role in salt intake. Therefore, due to lack of budget, we selected the most important polymorphism which could have role and other variants will be studied in the future. Genotyping design of this variant is based on reported variant in dbSNP database.

### Genotyping analysis

DNA was isolated from peripheral blood lymphocytes using the standard salting out method ^29^. Genotyping was carried out using ARMS method for allelic discrimination, and validated by capillary sequencing (AB3730, Applied Biosystems). Primers and annealing temperature used in the study are listed in Supplementary Appendix 1. The reaction details are as follows: PCR using an Eppendorf gradient type master cycler (Eppendorf, Germany) with a total volume of 16 µL (8 µL Taq PCR Master Mix, 0.4 µL each outer primer (10 mM), 0.2 µL each inner primer (10 mM), 1 µL genomic DNA and 3.2 µL H2O). After initial denaturation (95 °C 3 min) 30 cycles (95 °C 45 s, annealing temperature (Supplementary Appendix 1) 45 s, 72 °C 15 s) of amplification were performed, followed by an extension (72 °C 15 s) and a final elongation step (72 °C 1 min).

### Statistical analysis

Kolmogorov–Smirnov test was used for assessing the data distribution in all datasets. Data were reported as mean ± standard deviation (SD) for quantitative variables and number (percentage) for qualitative variables. A chi-square (χ^2^) test was performed to determine whether distributions of the genotypes of the study polymorphisms were in Hardy–Weinberg equilibrium. One-way analysis of variance (ANOVA) and then Bonferroni as post hoc method (for correcting significance level due to multiple tests) were utilized to compare means in variables with normal distribution. Kruskal–Wallis test and then Mann–Whitney test as post hoc method were used to evaluate means of continuous variables across different genotypes of each SNP, when the assumptions of one-way analysis of variance were not met. Distribution of categorical variables across quartiles of different genotypes of each SNP were assessed using Chi-square test. We considered 2-tailed P values of less than 0.05 to be statistically significant. Analyses were conducted using SPSS statistical software version 19.0 for windows (SPSS Inc., Chicago, USA).

### Ethics approval and consent to participate

This study was funded by National Institute for Medical Research Development (NIMAD, Grant Number 977549). Written informed consents were obtained from adult participants and the parents of children.

## Results

Totally, 116 healthy adults (56% male and 44% female) were chosen for the study. Participants’ characteristics are summarized in Table 1. Participants had a mean age of 35.4 ± 7.7 years, BMI of 27.3 ± 4.9 kg/m^2^, total physical activity of 774.1 ± 667.3 METs minute per week, SBP of 122.6 ± 16.3 mmHg, DBP of 80.3 ± 14.7 mmHg and 11.2% of subjects were currently smokers. The 24UNa mean as a surrogate of sodium intake was 4651.0 ± 1647.7 mg/day. Table 2 shows the dietary intake in participants based on sex. The mean energy intake was 2080.5 ± 1656.5 kcal/day and the percentage of energy from carbohydrate, protein, total fat, saturated fatty acid (SFA), monounsaturated fatty acid (MUFA) and polyunsaturated fatty acid (PUFA) were 54.8 ± 8.7, 15.4 ± 2.5, 30.6 ± 7.4, 12.4 ± 5.9, 8.9 ± 2.5 and 8.2 ± 3.1, respectively and the mean fiber intake was 22.2 ± 10.7. The genotypic frequencies of the polymorphisms of rs239345 in the *SCNN1B* and rs4790151, rs224534 and rs8065080 in the*TRPV1* in the participants of our study were consistent with the Hardy–Weinberg equilibrium (Table 3). Table 4 shows genotypes and alleles distributions of the SNP rs239345 in the *SCNN1B* and SNPs rs4790151, rs224534, rs8065080 in the*TRPV1* genes.

**Table 1:** Basic characteristics of participants based on sex in 116 randomly selected adult participants of Isfahan city, Iran.

Characteristics	Total N = 116	Male N = 65	Female N = 51
Age (year)	35.7 ± 7.7	35.8 ± 7.1	36.1 ± 9.0
Body mass index (kg/m^2^)	27.3 ± 4.9	26.5 ± 4.0	28.8 ± 6.1
Waist circumference (cm)	100.4 ± 12.4	100.5 ± 12.3	100.1 ± 12.8
Total physical activity (METs/minute per week)	774.1 ± 667.3	869.6 ± 690.4	652.4 ± 537.5
Systolic blood pressure (mmHg)	122.6 ± 16.3	121.6 ± 16.0	125.5 ± 17.7
Diastolic blood pressure (mmHg)	80.3 ± 14.7	81.0 ± 13.1	80.0 ± 18.3
Fasting blood glucose (mg/dL)	86.0 ± 17.2	86.5 ± 18.9	84.9 ± 15.5
Total cholesterol (mg/dL)	189.2 ± 40.4	188.8 ± 35.7	192.9 ± 49.2
Triglyceride (mg/dL)	191.2 ± 113.7	194.5 ± 103.2	191.0 ± 137.3
Low density lipoprotein (mg/dL)	102.8 ± 24.9	102.2 ± 23.6	105.5 ± 28.4
High density lipoprotein (mg/dL)	47.0 ± 10.2	47.4 ± 9.8	46.7 ± 11.3
24-h urine sodium (mg/day)	4651.0 ± 1647.7	4223.2 ± 1718.5	4532.0 ± 15.93.9
Urine creatinine (mg/dL)	1813.2 ± 1087.5	2003.0 ± 1318.0	1445.9 ± 576.0
Urine volume (mL/day)	1197.2 ± 551.9	1351.3 ± 637.2	1033.6 ± 361.6
**Education n (%)**			
Illiterate and primary school	7 (6.1)	2 (3.1)	5 (9.8)
Guidance and high school	67 (57.7)	36 (55.4)	31 (60.8)
University	42 (36.2)	27 (41.5)	15 (29.4)
Current smoker n (%)	13 (11.2)	13 (20)	0 (0)

**Table 2:** Mena and standard deviation of dietary energy and nutrients intake of participants based on sex in 116 randomly selected adults participants of Isfahan city, Iran.

Nutrient	Total N = 116	Male N = 65	Female N = 51
Energy (Kcal/day)	2080.5 ± 1656.5	2117.6 ± 1083.5	2019.7 ± 2327.7
Carbohydrate (% of energy)	54.8 ± 8.7	55.3 ± 8.6	54.1 ± 8.9
Protein (% of energy)	15.4 ± 2.5	15.1 ± 2.5	15.7 ± 2.5
Total fat (% of energy)	30.6 ± 7.4	30.9 ± 8.0	30.0 ± 6.3
SFA (% of energy)	12.4 ± 5.9	12.4 ± 6.5	12.4 ± 4.9
MUFA (% of energy)	8.9 ± 2.5	8.9 ± 2.6	9.1 ± 2.4
PUFA (% of energy)	8.2 ± 3.1	8.1 ± 3.2	8.3 ± 3.0
Fiber (g/day)	22.2 ± 10.7	22.7 ± 11.4	21.4 ± 9.7

**Table 3:** Conformation of the genotypes distributions of the study single nucleotide polymorphisms with Hardy–Weinberg equilibrium.

		Hardy–Weinberg	Our study	P value
rs239345	T allele	0.74	0.646	< 0.001
	A allele	0.26	0.354	0.011
rs8065080	T allele	0.683	0.849	< 0.001
	C allele	0.317	0.151	< 0.001
rs224534	C allele	0.63	0.6	0.028
	T allele	0.37	0.4	0.028
rs4790151	C allele	0.79	0.63	< 0.001
	T allele	0.21	0.36	< 0.001

**Table 4:** The frequency of genotypes and alleles distributions of the study single nucleotide polymorphisms in the ENaC and TRPV1 genes in 116 randomly selected adult participants of Isfahan city, Iran.

	rs8065080			rs239345			rs224534			rs4790151		
		n	%		n	%		n	%		n	%
Genotype frequencies	TT	83	71.6	TT	53	45.7	CC	38	32.8	CC	37	31.9
	TC	31	26.7	TA	44	38	CT	62	53.4	CT	73	63
	CC	2	1.7	AA	19	16.3	TT	16	13.8	TT	6	5.1
Allele frequencies	T	197	84.9	T	150	64.6	C	138	59.5	C	147	63.4
	C	35	15.1	A	82	35.4	T	94	40.5	T	85	36.6

### Single nucleotide polymorphisms and their association with sodium sources intake

The mean sodium intake from its major sources according to genotypes of studied SNPs are shown in Table 5. The intake of added salt in subjects with AA genotype (SNP rs239345, *P* = 0.010) was significantly higher than those with TT genotype. However, there was no significant association between various genotypes and mean intake of dietary sources of sodium in other SNPs.

**Table 5:** The mean and standard deviations of sodium intake from different food sources according to different genotypes of the study single nucleotide polymorphism in 116 randomly selected adult participants of Isfahan city, Iran.

Sodium sources (g/day)	rs239345			P^*1^	rs4790151			P^2^	rs8065080			P	rs224534			P^2^
	AA	AT	TT		CC	CT	TT		TT	CT	CC		CC	CT	TT	
Added salt	2507.5 (623.6)	2352.3 (976.7)	2001.3 (899.7)^1^	0.010	2704.9 (668.7)	2352.0 (917.2)	2588.8 (1545.9)	0.228	2234.5 (912.7)	2478.3 (947.2)	1722.2 (864.2)	0.318	2457.7 (928.3)	2228.8 (931.3)	2119.2 (870.2)	0.358
Bread	143.0 (126.2)	161.9 (146.7)	202.2 (169.0)	0.317	163.9 (163.7)	188.0 (155.3)	149.1 (89.3)	0.714	175.0 (153.9)	168.9 (155.3)	382.7 (59.5)	0.165	168.0 (154.7)	179.6 (137.3)	193.6 (227.7)	0.882
Cheese	14.9 (13.5)	14.9 (14.9)	12.7 (12.1)	0.724	13.5 (12.5)	13.6 (14.0)	18.5 (10.8)	0.728	14.8 (12.6)	12.3 (15.1)	4.0 (5.6)	0.416	13.8 (12.5)	13.6 (13.9)	14.9 (13.6)	0.955
Other salty dairies	121.7 (106.1)	101.3 (108.2)	101.6 (98.7)	0.767	131.9 (132.9)	91.5 (79.3)	83.5 (94.7)	0.178	103.3 (94.17)	110.4 (122.9)	90.7 (116.1)	0.936	133.3 (120.2)	94.4 (96.9)	78.9 (59.3)	0.159
Fast food	10.8 (17.9)	15.0 (22.4)	12.8 (18.5)	0.768	18.3 (22.0)	11.1 (17.9)	6 (12.7)	0.109	15.3 (21.9)	8.2 (13.2)	13.6 (8.6)	0.290	11.8 (15.9)	14.6 (22.0)	10.5 (19.2)	0.731
Salty snack	3.5 (6.7)	3.3 (6.3)	3.6 (7.7)	0.978	5.0 (8.6)	2.6 (5.8)	3.2 (7.1)	0.298	3.3 (7.4)	3.3 (6.0)	10.6 (0.0)	0.351	3.8 (8.8)	3.4 (6.4)	2.7 (3.7)	0.889
Canned food	2.2 (6.6)	3.7 (11.1)	4.1 (14.7)	0.863	0.9 (4.0)	4.6 (13.9)	10.8 (24.2)	0.163	2.54 (10.5)	6.5 (15.8)	0.0 (0.0)	0.324	2.5 (10.6)	4.5 (13.8)	2.9 (10.1)	0.770
Salty nut and seed	10.6 (21.4)	13.1 (18.0)	11.0 (21.9)	0.913	11.8 (21.7)	12.9 (20.3)	2.8 (2.7)	0.569	9.7 (16.6)	12.6 (21.0)	17.7 (12.9)	0.235	7.9 (12.0)	15.2 (22.1)	8.9 (27.5)	0.247
Sauce	7.7 (7.5)	3.2 (3.1)	3.5 (3.3)	0.140	5.9 (12.8)	3.3 (4.0)	2.5 (2.7)	0.334	3.1 (3.3)	6.3 (14.1)	8.5 (0.0)	0.169	3.2 (3.5)	4.9 (10.6)	3.4 (3.7)	0.607

Kruskal–Wallis test.

^*^P P value.

^1^P value = Significant difference with AA genotype: Post hoc analysis.

^2^P value = Significant difference with CC genotype: Post hoc analysis.

### Single nucleotide polymorphisms and their association with salt intake and blood pressure

The comparison of mean sodium intake, SBP and DBP between related genotypes of different SNPs are shown in Table 6. There were significant differences between AA and TT genotypes of SNP rs239345 for sodium intake (4808.4 ± 824.4 mg/day vs. 4043.5 ± 989.3 mg/day; *P* = 0.004) and DBP (83.6 ± 8.5 mmHg vs. 77.3 ± 7.3 mmHg; P = 0.011). In addition, the mean sodium intake and DBP was significantly higher in A vs. T allele in SNP rs239345 (P = 0.035). The sodium intake was higher in the CC genotype of rs224534 in the *TRPV1* than the TT genotype (4633.3 ± 793.5 mg vs. 3767.0 ± 713.7 mg/day/day; P = 0.012). It was also higher in C than T allel in SNP rs224534 (P = 0.029). There was no relationship between all SNPs and SBP, also SNPs including rs224534, rs4790151 and rs8065080 with DBP. Furthermore, non-significant differences were found in dietary sodium intake, with all genotypes of rs4790151 and rs8065080 SNPs in the *TRPV1* gene.

**Table 6:** The mean and standard deviations of sodium intake and blood pressure according to different genotypes of the study single nucleotide polymorphisms in 116 randomly selected adult participants of Isfahan city, Iran.

Variables	rs239345			P^*1^	rs4790151			P^2^	rs8065080			P^2^	rs224534			P
	AA	AT	TT		CC	CT	TT		TT	CT	CC		CC	CT	TT	
Sodium intake (mg/day)	4808.4 (824.4)	4344.8 (979.4)	4043.2^1^ (989.3)	0.004	4148.5 (967.9)	4461.5 (1015.1)	4564.8 (643.4)	0.514	4471.7 (1009.9)	4075.2 (918.7)	4351.1 (194.4)	0.171	4633.3 (793.5)	4356.2 (1097.0)	3767.0^2^ (713.7)	0.012
SBP (mmHg)	123.9 (15.4)	121.9 (16.2)	120.6 (11.3)	0.670	124.4 (13.7)	120.3 (14.5)	120.5 (6.7)	0.306	122.7 (14.1)	119.0 (14.1)	120.5 (0.7)	0.469	122.8 (14.3)	121.3 (14.1)	120.3 (13.7)	0.805
DBP (mmHg)	83.6 (8.5)	80.9 (9.1)	77.3 (7.3)	0.011	80.7 (9.0)	79.1 (8.4)	82.0 7.1)	0.798	80.0 (8.7)	79.4 (8.1)	77.5 (6.4)	0.876	80.8 (7.7)	80.4 (8.7)	75.1 (8.4)	0.064

Analysis of variance test.

^*^P P value.

^1^Significant difference with AA genotype: Post hoc analysis.

^2^Significant difference with CC genotype: Post hoc analysis.

## Discussion

The current study examined the association between variations in taste detection genes and sodium intake as well as BP for the first time in Iran and Eastern Mediterranean region. The findings of this study showed that among four SNPs which were studied, only two SNPs including rs239345 and rs224534 were significantly related to sodium intake and only rs239345 was involved in DBP level.

Regulation of salt or sodium intake is partly due to genes variation related to homeostatic sodium regulation and to hedonic responses to the salt taste ^6,30^. In this study, it was shown that individuals with *SCNN1B* gene polymorphism of AA/AT, rs239345, coded for SCNN1B and the *TRPV1* gene polymorphism of CC, rs224534 had higher sodium intake. Moreover, individuals with AA genotype of SNP rs239345 had higher consumption of added salt, as one of main source of salt intake ^31^. In addition, individuals with AA genotype of SNP rs239345 had higher DBP level than those with TT genotype. Cheilat’s cross-sectional study on 70 families including children and parents in Canada showed that these SNPs were involved in sodium intake, BP and CVD, as A allele carriers had higher sodium intake and DBP. However, as Cheilat’s study read the reverse strand, it found this association with T allele ^32^. Moreover, Chamoun’s study on Canadian young adults and preschool children illustrated associations between rs4790522 and rs222745 SNPs in the *TRPV1* salt taste receptor gene and salt taste sensitivity in young adults and salt taste preference in children ^33^. Pilic’s study among young Caucasians in the UK revealed that the subjects with AA genotype in SNP SLC4A5 rs7571842 had the highest increase in SBP and DBP; however, SNP rs10177833 (SLC4A5), rs239345 (SCNN1B) and rs8065080 (TRPV1) had no statistically significant effects on the BP response to dietary Na manipulation and with the increasing number of A alleles in SNP SLC4A5 rs10177833, sodium intake increased ^12^. In addition, Barragan’ study among Caucasians aged 18–80 in Spain indicated that those with AA genotype had the highest salty taste intensity rate ^13^. Conversely, the SNP rs239345 (SCNN1B) was not significantly associated with salt sensitivity or salt taste thresholds in Hungarian Roma and young Caucasian subjects ^11,12,17^.

It is well established that sodium consumption is associated with elevated BP in multiple populations ^34^. In the present study, in line with Cheilat’s study, it has been shown that high DBP belongs to the carriers of A allele. Genetic polymorphisms or acquired over-activity of the *ENaC* is accompanied with arterial hypertension ^35^, despite the fact that the link between sodium intake CVD events was controversial in subjects without hypertension ^36^. Research on the alpha subunit of *ENaC* suggests a potential implication on BP regulation in mice ^37^. Furthermore, the rs239345 polymorphism on the beta subunit of *ENaC*) may be associated with another SNP, perhaps, the same one that related to BP in the alpha subunit of the *ENaC* in rats ^38^.

The taste responses to salt can be obstructed by the *ENaC* blocker amiloride without similar effect on other taste manners in mice ^39^. However, this mechanism can inhibit about 20% of salt taste perception in humans. Thus, to some extent, salt taste responses are regulated by *ENaC* in humans ^40^. However, other markers related to sodium intake were studied including salt taste thresholds and learned responses ^17,41^.

Similar to our findings, Ferraris et al. implied that there were no significant associations between the rs8065080 SNP in *TRPV1* gene with an individual’s salt intake, SBP and DBP ^14^. However, the studies by Dias et al., Dioszegi et al. and Pilic et al., showed that T allele carriers perceived salt solutions significantly stronger than those homozygous for the C allele ^6,11,17^. An isoleucine (C) to valine (T) amino acid replacement (585 position of the TRPV1 protein, missense mutation) leads to rs8065080 (C > T) polymorphism. Contrary to our results, Pilic et al. ^17^ reported that TRPV1 rs8065080 T allele carriers had higher sodium intake than C allele carriers in a small sample of young predominantly Caucasian participants. The potential reason of these contradiction might be due to TRPV1 rs8065080 missense mutation and hence altering one amino acid ^42^. Moreover, age difference between the studies could be another reason, since age is a factor in phenotypical variance in genetic expression ^43^.

However, owing to the restricted research in the salt taste genotypes, the comparison of findings are limited, and therefore, highlighting the necessity of performing further well-designed studies.

### Strengths and limitations

To the best of our knowledge, it was the first genetic study on salt intake in the Eastern Mediterranean region. In addition, we examined sodium intake by the precise method of 24-h urine sodium measurement and food contribution with validated FFQ in our population. However, we also had some limitations including limited coverage of polymorphisms within the most important *SCNN1B* -associated gene and *TRPV1*, low sample size and lack of sequencing possibility for all samples and the fact that collecting a single 24-h urine was not enough to reflect a true customary intake. Although it has been proposed that some other SNPs might be associated with sodium intake, because of financial limitation, we did not examine them. Finally, not adjusting age, sex and BMI was another limitation. It might alter the association of salt intake and blood pressure with the SNPs.

## Conclusion

This study demonstrated that SNPs in the *SCNN1B* and *TRPV1* genes associated with sodium intake and BP level. Therefore, genetic variations can relate with salt intake and consequently may associate with hypertension and finally CVD risk in the Iranian population. Further studies with larger sample size are warranted to replicate these results in order to better understand the genetic basis for salt taste and hypertension risk and also examine the potential effect of interactions between the diverse SNPs with the valid urine collection method can be effective in accurate estimation of sodium intake.

## Supplementary Information

Supplementary Information.

## Data Availability

The authors confirm that the *data* supporting the findings of this study are available.

## Abbreviations

CVD: Cardiovascular disease
AS: Amiloride
AI: Amiloride-insensitive
SNP: Single nucleotide polymorphisms
BP: Blood pressure
ICRI: Isfahan Cardiovascular Research Institute
IPAQ: International Physical Activity Questionnaire
BMI: Body mass index
WC: Waist circumference
DBP: Diastolic blood pressure
SBP: Systolic blood pressure
FFQ: Food frequency questionnaire
IFCP: Iranian Food Consumption Program
FBG: Fasting blood glucose
24hUNa: 24-h urinary sodium
CR: Creatinine
24hUCr: 24-h urine creatinine

## References

[1] Mozaffarian D, Fahimi S, Singh GM, Micha R, Khatibzadeh S, Engell RE (2014). Global sodium consumption and death from cardiovascular causes. N. Engl. J. Med..

[2] Messerli FH, Hofstetter L, Syrogiannouli L, Rexhaj E, Siontis GC, Seiler C, Bangalore S (2021). Sodium intake, life expectancy, and all-cause mortality. Eur. Heart J..

[3] Grillo A, Salvi L, Coruzzi P, Salvi P, Parati G (2019). Sodium intake and hypertension. Nutrients.

[4] Bigiani A (2020). Salt taste, nutrition, and health. Nutrients.

[5] Yoshida R, Horio N, Murata Y, Yasumatsu K, Shigemura N, Ninomiya Y (2009). NaCl responsive taste cells in the mouse fungiform taste buds. Neuroscience.

[6] Dias AG, Rousseau D, Duizer L, Cockburn M, Chiu W, Nielsen D (2013). Genetic variation in putative salt taste receptors and salt taste perception in humans. Chem. Senses.

[7] Tapanee P, Tidwell DK, Schilling M, Peterson DG, Tolar-Peterson T (2021). Genetic variation in taste receptor genes (SCNN1B, TRPV1) and its correlation with the perception of saltiness in normotensive and hypertensive adults. Int. J. Hypertens.

[8] Bertino M, Beauchamp GK, Engelman K (1982). Long-term reduction in dietary sodium alters the taste of salt. Am. J. Clin. Nutr..

[9] Wise PM, Hansen JL, Reed DR, Breslin PA (2007). Twin study of the heritability of recognition thresholds for sour and salty taste. Chem. Senses.

[10] Mozaffarian D (2016). Dietary and policy priorities for cardiovascular disease, diabetes, and obesity: A comprehensive review. Circulation.

[11] Diószegi J, Kurshed AA, Pikó P, Kósa Z, Sándor J, Ádány R (2021). Association of single nucleotide polymorphisms with taste and food preferences of the Hungarian general and Roma populations. Appetite.

[12] Pilic L, Lubasinski NJ, Berk M, Ward D, Graham CA, Anastacio VD, King A, Mavrommatis Y (2020). The associations between genetics, salt taste perception and salt intake in young adults. Food Qual. Prefer..

[13] Barragán R, Coltell O, Portolés O, Asensio EM, Sorlí JV, Ortega-Azorín C, González JI, Sáiz C, Fernández-Carrión R, Ordovas JM, Corella D (2018). Bitter, sweet, salty, sour and umami taste perception decreases with age: Sex-specific analysis, modulation by genetic variants and taste-preference associations in 18- to 80-year-old subjects. Nutrients.

[14] Ferraris C, Turner A, Kaur K, Piper J, Veysey M, Lucock M, Beckett EL (2020). Salt taste genotype, dietary habits and biomarkers of health: No associations in an elderly cohort. Nutrients.

[15] DeSimone JA, Lyall V (2006). Taste receptors in the gastrointestinal tract III. Salty and sour taste: Sensing of sodium and protons by the tongue. Am. J. Physiol. Gastrointest. Liver Physiol..

[16] Gu X, Gu D, He J, Rao DC, Hixson JE, Chen J (2018). resequencing epithelial sodium channel genes identifies rare variants associated with blood pressure salt-sensitivity: The GenSalt study. Am. J. Hypertens..

[17] Pilic L, Mavrommatis Y (2018). Genetic predisposition to salt-sensitive normotension and its effects on salt taste perception and intake. Br. J. Nutr..

[18] Craig CL, Marshall AL, Sjöström M, Bauman AE, Booth ML, Ainsworth BE (2003). International physical activity questionnaire: 12-country reliability and validity. Med. Sci. Sports Exerc..

[19] Chobanian AV, Bakris GL, Black HR, Cushman WC, Green LA, Izzo JL (2003). Seventh report of the Joint National Committee on prevention, detection, evaluation, and treatment of high blood pressure. Hypertension.

[20] Mohammadifard N, Khosravi AR, Esmaillzadeh A, Feizi A, Abdollahi Z, Salehi F (2016). validation of simplified tools for assessment of sodium intake in Iranian population: Rationale, design and initial findings. Arch. Iran Med..

[21] Rafiei M, Boshtam M, Marandi A (2002). The Iranian food consumption program (IFCP): A unique nutritional sotware in Iran. Iran. J. Public Health.

[22] Dorosti Motlagh AR, Tabatabaei M (2007). Iranain Food Composition Table 1.

[23] Mohammadifard N, Khaledifar A, Khosravi A, Nouri F, Pourmoghadas A, Feizi A (2017). Dietary sodium and potassium intake and their association with blood pressure in a non-hypertensive Iranian adult population: Isfahan salt study. Nutr. Diet.

[24] Hedayati SS, Minhajuddin AT, Ijaz A, Moe OW, Elsayed EF, Reilly RF (2012). Association of urinary sodium/potassium ratio with blood pressure: Sex and racial differences. Clin. J. Am. Soc. Nephrol..

[25] Wang CY, Cogswell ME, Loria CM, Chen TC, Pfeiffer CM, Swanson CA (2013). Urinary excretion of sodium, potassium, and chloride, but not iodine, varies by timing of collection in a 24-hour calibration study. J. Nutr..

[26] Yakubova A, Davidyuk Y, Tohka J, Khayrutdinova O, Kudryavtsev I, Nurkhametova D (2021). Searching for predictors of migraine chronification: A pilot study of 1911A>G polymorphism of TRPV1 gene in episodic versus chronic migraine. J. Mol. Neurosci..

[27] González-Mercado A, Magaña-Torres MT, Sánchez-López JY, Ríos-Silva M, Ibarra-Cortés B, Trujillo X (2020). The relationship of single nucleotide polymorphisms in the TRPV1 gene with lipid profile, glucose, and blood pressure in Mexican population. Genet. Test Mol. Biomark..

[28] Cantero-Recasens G, Gonzalez JR, Fandos C, Duran-Tauleria E, Smit LA, Kauffmann F (2010). Loss of function of transient receptor potential vanilloid 1 (TRPV1) genetic variant is associated with lower risk of active childhood asthma. J. Biol. Chem..

[29] Koshy L, Anju AL, Harikrishnan S, Kutty VR, Jissa VT, Kurikesu I, Jayachandran P (2017). Evaluating genomic DNA extraction methods from human whole blood using endpoint and real-time PCR assays. Mol. Biol. Rep..

[30] Zhao Q, Gu D, Hixson JE, Liu DP, Rao DC, Jaquish CE (2011). Common variants in epithelial sodium channel genes contribute to salt sensitivity of blood pressure: The GenSalt study. Circ. Cardiovasc. Genet..

[31] Mohammadifard N, Mahdavi A, Khosravi A, Esmaillzadeh A, Feizi A, Saarfzadegan N (2021). Salt intake and its sources in children, adolescents and adults in Isfahan, Islamic Republic of Iran. East Mediterr. Health J..

[32] Chleilat, F. *Genetic Variation in Salt Taste Receptors Impact Salt Intake, Blood Pressure and Cardiovascular Disease Risk Factors in the Guelph Family Health Study.* http://hdl.handle.net/10214/10069 (2016). (Accessed 12 February 2020).

[33] Chamoun E, Carroll NA, Duizer LM, Qi W, Feng Z, Darlington G (2018). The relationship between single nucleotide polymorphisms in taste receptor genes, taste function and dietary intake in preschool-aged children and adults in the Guelph Family Health Study. Nutrients.

[34] Mente A, O'Donnell MJ, Rangarajan S, McQueen MJ, Poirier P, Wielgosz A (2014). Association of urinary sodium and potassium excretion with blood pressure. New Engl. J. Med..

[35] Bubien JK (2010). Epithelial Na+ channel (ENaC), hormones, and hypertension. J. Biol. Chem..

[36] Mente A, O’Donnell M, Rangarajan S, Dagenais G, Lear S, McQueen M (2016). Associations of urinary sodium excretion with cardiovascular events in individuals with and without hypertension: A pooled analysis of data from four studies. Lancet.

[37] Shigemura N, Ohkuri T, Sadamitsu C, Yasumatsu K, Yoshida R, Beauchamp GK (2008). Amiloride-sensitive NaCl taste responses are associated with genetic variation of ENaC alpha-subunit in mice. Am. J. Physiol. Regul. Integr. Comp. Physiol..

[38] Shehata MF (2010). The alternatively spliced form "b" of the epithelial sodium channel α subunit (α ENaC): Any prior evidence of its existence?. Clin. Med Insights Cardiol..

[39] Stewart RE, DeSimone JA, Hill DL (1997). New perspectives in a gustatory physiology: Transduction, development, and plasticity. Am. J. Physiol..

[40] Bigiani A (2020). Does ENaC work as sodium taste receptor in humans?. Nutrients.

[41] Gilman TL, George CM, Andrade MA, Mitchell NC, Toney GM, Daws LC (2019). High salt intake lowers behavioral inhibition. Front. Behav. Neurosci..

[42] National Center for Biotechnology Information. *Data Base of Single Nucleotide Polymorphisms (dbSNP)*. https://www.ncbi.nlm.nih.gov/snp/ (Accessed 20 January 2022).

[43] Viñuela A, Brown AA, Buil A, Tsai PC, Davies MN, Bell JT (2018). Age-dependent changes in mean and variance of gene expression across tissues in a twin cohort. Hum. Mol. Gen..

