# Supplementary material for: Genetic variation in salt taste receptors impact salt intake and blood pressure
Source: Sci Rep. 2023 Mar 10;13:4037. doi: 10.1038/s41598-022-23827-0 (PMC10006406; doi:10.1038/s41598-022-23827-0)
Supplement: Supplementary file 1 — Supplementary Information. [file 41598_2022_23827_MOESM1_ESM.docx]

**Appendix1:**

| **Name** | **direction** | **Primer sequences** | **Product size (bp)** | **Annealing temperature** |
| --- | --- | --- | --- | --- |
| **Rs8065080** | Forward outer | CCGACCAGAACAAAGGCTATG | 246 | 60 |
|  | Reverse inner | GAAAACCCGAACAAGAAGACGAT |  |  |
|  | Forward inner | GCCGTTTCATGTTTGTCTACG | 200 |  |
|  | Reverse outer | CTCATTTCAGTGTGTCCTCTGTC |  |  |
| **Rs239345** | Forward outer | GTAAAGAGACAAGGTGGCATGTG | 210 | 62 |
|  | Reverse inner | AACAGCTTTACCACAAGAGCTGA |  |  |
|  | Forward inner | TGTTCTTCCTTGCTTTGCTTCCA | 155 |  |
|  | Reverse outer | CACACGTGTGCACAGTTTAAGGATG |  |  |
| **Rs224534** | Forward outer | GGCGCTCCCCTAATAACACACTGT | 202 | 64 |
|  | Reverse inner | TCTCCAGTAACTCGGAAATAGTCTCCAA |  |  |
|  | Forward inner | GCCTCCCTTTAAGATGGAAAAAAC | 249 |  |
|  | Reverse outer | CTGAAGGGCTCTCATCTTCACCT |  |  |
| **rs4790151** | Forward outer | CAGGCTGGTCTCAAACTCCT | 279 | 61 |
|  | Reverse inner | TTCAGATCCAGGTGCCATAA |  |  |
|  | Forward inner | CTAAGAGAGACCTAATAGGTGTGTGC | 246 |  |
|  | Reverse outer | CCTCAGAGGTCCATTTTGCT |  |  |
